# Glyphosate-Induced Phosphonatase Operons in Soil Bacteria of the Genus *Achromobacter*

**DOI:** 10.3390/ijms25126409

**Published:** 2024-06-10

**Authors:** Dmitry O. Epiktetov, Alexey V. Sviridov, Sergey V. Tarlachkov, Tatyana V. Shushkova, Ilya Yu. Toropygin, Alexey A. Leontievsky

**Affiliations:** 1 G.K. Skryabin Institute of Biochemistry and Physiology of Microorganisms, Federal Research Center “Pushchino Scientific Center for Biological Research of the Russian Academy of Sciences”, Russian Academy of Sciences, 5 Prosp. Nauki, 142290 Pushchino, Russia; epiktetoff@pbcras.ru (D.O.E.); sergey@tarlachkov.ru (S.V.T.); tanya_vsh@rambler.ru (T.V.S.); leont@ibpm.pushchino.ru (A.A.L.); 2 Branch of Shemyakin—Ovchinnikov Institute of Bioorganic Chemistry, Russian Academy of Sciences, Puschino, 142290 Moscow, Russia; 3 V.N. Orekhovich Research Institute of Biomedical Chemistry, Bld. 8, 10 Pogodinskaya Str., 119121 Moscow, Russia; toropygin@rambler.ru

**Keywords:** organophosphonates, glyphosate, phosphonatase, catabolism, oxygenases, operons, protein purification, enzyme kinetics

## Abstract

*Achromobacter insolitus* and *Achromobacter aegrifaciens*, bacterial degraders of the herbicide glyphosate, were found to induce phosphonatase (phosphonoacetaldehyde hydrolase, EC 3.11.1.1) when grown on minimal media with glyphosate as the sole source of phosphorus. The phosphonatases of the strains were purified to an electrophoretically homogeneous state and characterized. The enzymes differed in their kinetic characteristics and some other parameters from the previously described phosphonatases. The phosphonatase of *A. insolitus* was first revealed to separate into two stable forms, which had similar kinetic characteristics but interacted differently with affinity and ion-exchange resins. The genomes of the investigated bacteria were sequenced. The phosphonatase genes were identified, and their context was determined: the bacteria were shown to have gene clusters, which, besides the phosphonatase operon, included genes for LysR-type transcription activator (substrate sensor) and putative iron-containing oxygenase PhnHD homologous to monooxygenases PhnY and TmpB of marine organophosphonate degraders. Genes of 2-aminoethylphosphonate aminotransferase (PhnW, EC 2.6.1.37) were absent in the achromobacterial phosphonatase operons; instead, we revealed the presence of genes encoding the putative flavin oxidase HpnW. In silico simulation showed 1-hydroxy-2-aminoethylphosphonate to be the most likely substrate of the new monooxygenase, and a number of glycine derivatives structurally similar to glyphosate to be substrates of flavin oxidase.

## 1. Introduction

**Figure 1:**
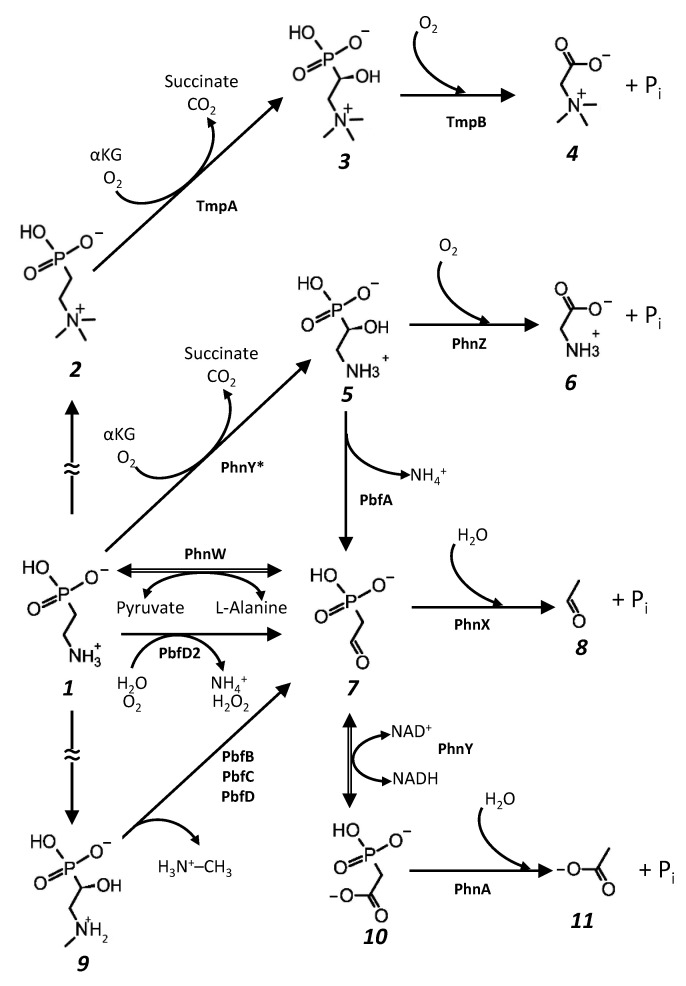
A diagram of the known 2-aminoethylphosphonic acid (2-AEP) degradation pathways: 1, 2-AEP; 2, 2-trimethylammonium ethylphosphonic acid; 3, HTAEP; 4, betaine; 5, HAEP; 6, glycine; 7, phosphonoacetaldehyde (PAA); 8, acetaldehyde; 9, *N*-methyl aminoethylphosphonic acid (MAEP); 10, phosphonoacetate; 11, acetic acid; P_i_, orthophosphate. Unidirectional arrows indicate irreversible reactions; bidirectional arrows indicate reversible reactions.

Organophosphonates (OP) are organic phosphorus compounds containing a direct covalent carbon–phosphorus C–P^3+^ bond (C–P bond) resistant to chemical and enzymatic hydrolysis. Recent research has shown the widespread occurrence of OP in wildlife. In some marine ecosystems, OP account for the bulk of bioavailable phosphorus, thus playing a significant role in the biological cycle of this element [1,2,3]. Large volumes of OP also enter the environment as a result of human activities as chelating additives and pesticides. Glyphosate (N-phosphonomethylglycine, GP) and the product of its primary degradation—aminomethylphosphonic acid (AMPA)—are among the most widespread phosphonate xenobiotics. Both natural and anthropogenic OP are metabolized by various microorganisms, primarily bacteria [1,2]. As a rule, the C–P bond is not cleaved by the known phosphatases and phosphoesterases. Specialized enzyme systems are required to utilize OP as sources of phosphorus, such as multi-protein C–P lyase complex and phosphonoacetaldehyde hydrolase or phosphonatase (PhnX, EC 3.11.1.1). The prevalence of these systems in the microbiota serves as a marker of the presence of significant amounts of OP in a given ecosystem [4]. Phosphonatase is also called a key enzyme of natural OP catabolism because it provides rapid hydrolysis of phosphonoacetaldehyde (PAA, Figure 1, *7*), a central metabolite of the pathways of OP synthesis and decomposition in bacteria [2,3].

Phosphonatase was discovered more than 50 years ago in a culture of *Bacillus cereus* capable of utilizing 2-aminoethylphosphonic acid (2-AEP, ciliatine, Figure 1, *1*) as the sole source of phosphorus [5,6] and is considered a well-investigated enzyme. The phosphonatase pathway of 2-AEP degradation implies the involvement of 2-AEP:pyruvate aminotransferase (2-AEP transaminase, PhnW, EC 2.6.1.37), whose products are PAA and alanine [7]. The mechanism of cleavage of the C–P bond in PAA by phosphonatase includes the formation of an intermediate Schiff base between the lysine residue and the carbonyl group of phosphonoacetaldehyde in the active center of the enzyme formed by two domains—cap and core—connected by a flexible linker [8,9].

**Figure 2:**
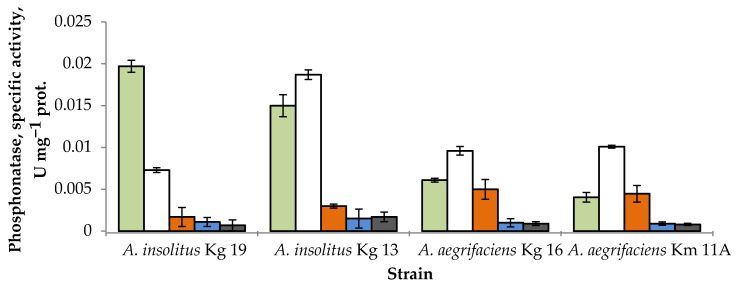
Phosphonatase activity in cell-free extracts of OP-degrading bacteria at the uptake of various sources of phosphorus designated by colors: green, GP; white, phosphorus starvation; orange, 2-AEP; blue, MPA; grey, P_i_. Vertical bars represent confidence intervals at a confidence level of 95%. Protein concentrations in cell-free extracts: *A. insolitus* Kg 19, 7.6 mg mL^−1^; *A. insolitus* Kg 13, 11.7 mg mL^−1^; *A. aegrifaciens* Kg 16, 6.6 mg mL^−1^; *A. aegrifaciens* Km 11A, 5.0 mg mL^−1^.

**Figure 3:**
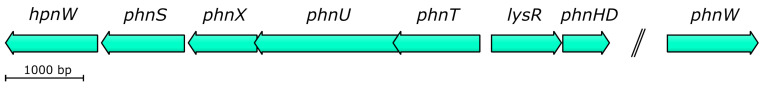
Organization of the phosphonatase gene clusters in *A. insolitus* Kg 19 and *A. aegrifaciens* Kg 16. Arrows designate open reading frames. *phnSTU* encode OP transporter components: *phnX* —phosphonatase, and *phnW*—2-AEP transaminase; *lysR*—transcriptional regulator; *hpnW* and *phnHD*—genes of putative enzymes of unknown function.

Recently, increased attention has been paid to the role of phosphonatase in the pathways of the synthesis and decomposition of natural compounds as part of the global phosphorus cycle [3,4,10]. New enzymes relating the degradation of 2-AEP and PAA to the catabolism of 1-hydroxy-2-aminoethylphosphonic acid (HAEP, Figure 1, *5*) have been found in a number of marine prokaryotes. Thus, oxygenase PhnZ containing two iron atoms in oxidation states 2+ and 3+ is distinguished by a novel oxidative mechanism of cleaving the C–P bond in HAEP. In turn, HAEP is formed by oxidation of 2-AEP by Fe^2+^-dioxygenase PhnY* [11]. Enzyme pairs, homologous to PhnY* and PhnZ but with different functions, have been discovered recently in *Leisingera caerulea* and *Gimesia maris*, which possessed, correspondingly, the TmpA/TmpB pair as a pathway of degradation of 1-hydroxy-2-trimethylammonium ethylphosphonic acid (HTAEP, Figure 1, *2*, *3*), and *Gm*PhnY*/*Gm*PhnZ as a novel mechanism of methylphosphonic acid (MPA) breakdown [12,13]. Two former pathways were present in bacteria devoid of phosphonatase and served as an alternative method for the disposal of 2-AEP and its alkyl derivatives. However, novel lyases and oxidoreductases, PbfA, PbfB, PbfC, and PbfD, for which genes have been detected within phosphonatase gene clusters in certain marine bacteria, can facilitate HAEP, 2-AEP, and *N*-methyl aminoethylphosphonic acid (MAEP, Figure 1, *9*) transformation into PAA, indicating that phosphonatase may actually serve as a terminal hydrolase for processes of decomposition of multiple hydroxylated and methylated 2-AEP derivates [10,14].

The assumption that phosphonatase could take part in the catabolism of GP by hydrolyzing phosphonoformaldehyde (PFA, formylphosphonate), a putative product of AMPA transamination, has been discussed in some works [15,16]. While narrow substrate specificity of the enzyme [17,18,19] contradicts this hypothesis and direct studies of the matter are hampered by the commercial inaccessibility and low stability of PFA [20,21], there are some data hinting at an interconnection between GP, AMPA, and the phosphonatase pathway. Induction of 2-AEP transport systems in *B. cereus* in the presence of AMPA was noted in the earliest works [5], while *Pseudomonas* sp. SG-1 demonstrated an activation of GP uptake upon introduction of pyridoxal-5′-phosphate and pyruvate into the medium [22]. Mutant strains of *Streptomyces viridochromogenes*, lacking the aldehyde dehydrogenase PhpJ that putatively oxidizes PFA, accumulate AMPA, indicating the existence of a transaminase specific to PFA [23]. Finally, the effect of increased phosphonatase production during the utilization of GP as the sole source of phosphorus was revealed by us in the soil strain *Ochrobactrum anthropi* GPK 3, which degraded the herbicide by its conversion to AMPA, followed by complete intracellular utilization [24].

The phosphonatase gene *phnX* and the transaminate gene *phnW*, together with the genes of other proteins involved in the utilization of 2-AEP, form a cluster representing an operon with a single promoter. *Salmonella typhimurium* LT2 has a seven-cistron cluster, *phnRSTUVWX*, comprising 2-AEP transmembrane transporter genes *phnSTUV* and the transcription regulator gene *phnR* [25]. A similar organization of the phosphonatase operon is described for various bacteria in modern publications on the subject [3,10]. In some cases, instead of the gene *phnX*, the genes of PAA dehydrogenase (*phnY*) and phosphonoacetate hydrolase (*phnA*) may be present, providing two-stage cleavage of PAA (Figure 1, *10*, *11*) [3].

Several mechanisms of phosphonatase synthesis regulation in bacteria are known. In the first case, the enzyme expression is under control of the PhoB/PhoR regulatory system. At low levels of P_i_, PhoB protein binds to the 18bp sequence (the *pho* box) in the promoter region of the phosphonatase operon and triggers transcription [26]. Certain mutations can lead to the emergence of bacteria with constitutive phosphonatase [6,25].

The second mechanism of regulation, revealed in strains of *P. putida* NG2 and *P. aeruginosa* PAO1, involves the induction of the operon by 2-AEP. The sensor protein belonging to the LysR transcription regulator family specifically interacts with 2-AEP, binds to the promoter region of the operon, and activates transcription. The *LysR* gene is usually found in close proximity to the phosphonatase operon, often as part of a single cluster. Interestingly, the substrate of phosphonatase proper (PAA) does not bind to LysR and cannot serve as the enzyme synthesis inducer [1,27].

Finally, in some bacteria, the PhoB/PhoR-controlled phosphonatase operon also includes the transcription regulator gene *phnR* [25]. Thus, the initial activation of the synthesis of 2-AEP catabolism enzymes caused by the depletion of P_i_ can be further precisely regulated by the presence and concentration of substrates and/or reaction products.

The expression of phosphonatase we observed previously, during the growth of *O. anthropi* GPK 3 culture on media with GP and AMPA in the absence of enzyme activity in cells grown on media with P_i_ or MPA [24], suggested the specific induction of the phosphonatase operon by the herbicide and its primary metabolite. This assumed the recognition of GP and AMPA as suitable phosphonatase pathway substrates. In this regard, it was of particular interest to search for bacteria with similar behavior among recently characterized, efficient GP-degrading cultures, also capable of growing in media with 2-AEP as the sole source of phosphorus [28]. Accordingly, the task of the present research was to study phosphonatases and their characteristics in bacteria that produce this enzyme while utilizing GP, as compared with those described in the literature, as well as to determine the structure and possible regulatory mechanisms of corresponding operons.

## 2. Results

### 2.1. Taxonomic Position of Bacteria under Study and Their Biodegrading Characteristics with Respect to OP

For the study, we chose four strains from the laboratory collection, also available from the All-Russian Collection of Microorganisms (VKM), which were distinguished with their stable physiological properties in long-term storage in minimal media with GP and were capable of effectively utilizing 2-AEP as the sole source of phosphorus, i.e., possessing an active phosphonatase system.

The previously performed multi-locus sequence typing of the *nrdA* gene region showed that strains Kg 16 and Km 11A belonged to the species *A. aegrifaciens*, and strains Kg 19 and Kg 13 to the species *A. insolitus*. Each pair of strains, however, belonged to different genogroups within its species [28].

All Achromobacter strains were capable of utilizing AMPA, MPA, GP, and 2-AEP as the sole sources of phosphorus, reaching high values of biomass when grown on media with these compounds (Table 1).

### 2.2. Dependence of Phosphonatase Activity in Cell-Free Extracts on the Source of Phosphorus in the Culture Medium and Selection of Research Objects

The determination of phosphonatase activity in cell-free extracts of the four bacterial strains after cultivation with various sources of phosphorus showed that in three cases, phosphorus starvation was the most potent factor in inducing the enzyme (Figure 2). Unexpectedly, in all bacteria, the phosphonatase activity after growth on GP exceeded that during the growth on 2-AEP, the natural substrate of the phosphonatase pathway. For strain *A. insolitus* Kg 19, GP served as the most potent inducer of the enzyme. In the presence of P_i_ or MPA, the studied strains demonstrated statistically similar results, exhibiting only a trace activity of phosphonatases, which is in agreement with previous data for *O. anthropi* [24]. 

Strains *A. insolitus* Kg 19 and *A. aegrifaciens* Kg 16 demonstrated higher yields of phosphonatase during growth on GP, as compared to *A. insolitus* 13 and *A. aegrifaciens* Km 11A, respectively, and were selected for further studies. Strain *A. aegrifaciens* Kg 16 was also considered to be of particular interest, as it had previously demonstrated unusual physiological properties in regard to GP utilization [29], which implied possible peculiarities in its OP metabolism enzymes.

### 2.3. Purification of Native Phosphonatases

Phosphonatases of wild-type strains had a very low yield, which made it difficult to measure enzyme activity and visualize proteins using electrophoresis. For this reason, instead of routine ultrasonic disintegration, we used extrusion of frozen biomass under pressure, which provided sufficient quantities of total protein for phosphonatase assay in the homogenate and for subsequent enzyme purification. After all stages of column chromatography, concentration of active preparations was required; after the last two stages, we had to perform silver staining of polyacrylamide gels.

The phosphonatases of two bacteria under study exhibited low stability during precipitation with ammonium sulfate or nonpolar solvents. For this reason, a purification scheme was proposed, including two anion exchange stages and gel filtration. DEAE-Toyopearl, due to its mechanical strength and high capacity, was convenient for primary separation of proteins of viscous cell-free extracts. It was also successfully used at the last stage of purification to get rid of minor ballast protein residues. The chosen technique made it possible to obtain electrophoretically homogeneous preparations of phosphonatases of *A. aegrifaciens* Kg 16 (hereinafter, PhnX16) and *A. insolitus* Kg 19 (hereinafter, PhnX19) with a yield of >10% and ~900–1200-fold purification (Table S1; Figure S1). In the latter strain, elution of phosphonatase from ion-exchange carriers occurred as two active peaks at NaCl concentrations of 0.16 M and 0.35 M, hereinafter referred to as PhnX19-I (~66% of eluted activity) and PhnX19-II (~34% of activity), respectively (Figure S2). After the initial separation, the preparations were further purified separately from each other (Figure S1A,B). At the last stage of purification, the preparations were eluted at the same NaCl concentrations as at the first stage, each in the form of a single active peak. PhnX16 was eluted in 0.15 M NaCl under the same conditions and did not separate into two active fractions.

During gel filtration, all three phosphonatase variants had very close retention volumes. Enzyme activity eluted within the range of 255–260 mL for PhnX16 and PhnX19-I and 250–260 mL for PhnX19-II, which corresponded to MWs of 61 ± 5 kDa and 68 ± 8 kDa, respectively. No enzyme activity was identified at a retention volume of 275 mL corresponding to MW of monomeric phosphonatase (30 kDa).

Purified preparations with phosphonatase activity, isolated from *A. insolitus* Kg 19, were subjected to trypsinolysis followed by MALDI-TOF analysis (Figure S3). Both protein variants corresponded to the theoretically predicted phosphonatase of *A. insolitus* presented in the NCBIprot database (https://www.ncbi.nlm.nih.gov/protein/OAD13647.1, accessed on 13 January 2021), with 79% coverage (score 180). The approach used did not reveal significant differences between PhnX19-I and PhnX19-II.

### 2.4. Making of Super-Producers of Phosphonatases and Purification of Recombinant Enzymes

During the cloning of the phosphonatase genes into the pET-22b vector at NdeI/XhoI sites, the *pelB* leader sequence present in the vector was eliminated, and constructs were obtained that provided overexpression of target proteins exactly corresponding in primary structure to wild-type phosphonatases, with the exception of a hexahistidine tag at the C-ends. Recombinant phosphonatases had cytoplasmic localization and retained their soluble form, without precipitation in the inclusion bodies.

Using affinity chromatography, we obtained electrophoretically homogeneous preparations of recombinant enzymes with yields of ~43% and ~95% for phosphonatases of Kg 16 (hereinafter, PhnX16-R) and Kg 19 (hereinafter, PhnX19-R), respectively (Table S2; Figure S4). Similar to PhnX19, in PhnX19-R the phosphonatase activity separated into two fractions at a ratio of ~20:80, eluted, respectively, at imidazole concentrations of 65 mM and 110 mM (Figure S5). The preparations were designated as PhnX19-RII (minor fraction) and PhnX19-RI (major fraction). For PhnX16-R, this phenomenon was not revealed: all phosphonatase activity was eluted at an imidazole concentration of 105 mM as a single peak.

### 2.5. Genome Sequencing and Analysis

An average of 4.3 × 10^7^ paired-end reads with length of 100 bp were obtained from the sequencing of *A. insolitus* Kg 19 and *A. aegrifaciens* Kg 16 strains. As a result, reads were assembled into scaffolds with greater than 600-fold coverage, where ~6100 genes were annotated for each assembly (Table S3). The genomes of *A. insolitus* Kg 19 and *A. aegrifaciens* Kg 16 were deposited in DDBJ/ENA/GenBank under the numbers JAALEO000000000 (https://www.ncbi.nlm.nih.gov/nuccore/JAALEO000000000.1, accessed on 17 July 2023) and JAVKVN000000000 (https://www.ncbi.nlm.nih.gov/nuccore/JAVKVN000000000.1, accessed on 17 October 2023), respectively.

The genes encoding the C–P lyase complex were identified in the genomes of both *A. insolitus* Kg 19 and *A. aegrifaciens* Kg 16 as two clusters, *phnCDE* and *phnNDuf1045phnMFGHIJKLM*, located within the same scaffold. In strain Kg 19, the gene *phnC* (G6N71_03490) contained a mutation with a reading frame shift, the expected result of which was the expression of the transmembrane phosphonate transporter PhnC in the form of a fragment of 145 amino acid residues instead of 278 in the wild-type protein. Genes of the PhoB/PhoR regulatory system were also present in the chromosomes of the investigated strains (NGT16298.1 and NGT16299.1, respectively, for *A. insolitus* Kg 19).

None of the bacteria showed the presence of *gox*, *gltA*, or *phnA* homologs.

The organization of the genes that ensure the functioning of the phosphonatase pathway in the strains under study differed from what was expected (Figure 3). Contrary to the literature data, the genes of 2-AEP transaminase, *phnW* (NGT14346.1 and MDR7943562.1 for *A. insolitus* Kg 19 and *A. aegrifaciens* Kg 16, respectively), and phosphonatase, *phnX* (NGT17078.1 and MDR7944343.1 for Kg 19 and Kg 16, respectively), were not located in the vicinity of each other and, in case of *A. insolitus* Kg 19, were within different scaffolds (JAALEO010000002.1 and JAALEO010000007.1, respectively). The chromosome of *A. insolitus* Kg 19 (scaffold JAALEO010000007.1) comprised an eight-cistron cluster, which included *phnX*, the *phnSTU* transport proteins’ genes (NGT17077.1, NGT17080.1, and NGT17079.1, respectively), and the *lysR* transcription regulator gene (NGT17081.1), as well as the genes of two putative proteins initially annotated as flavin oxidoreductase (NGT17076.1), hereinafter referred to as HpnW, and phosphohydrolase of the HDc superfamily (NGT17082.1), hereinafter referred to as PhnHD. Similar data were obtained by analyzing the genome of *A. aegrifaciens* Kg 16 (scaffolds JAVKVN010000001.1, MDR7944344.1, MDR7944341.1, MDR7944342.1, MDR7944340.1, MDR7944345.1, and MDR7944339.1, respectively).

### 2.6. Characterization of Native and Recombinant Phosphonatases

We determined the major kinetic and molecular characteristics for both recombinant and native phosphonatases of *A. insolitus* Kg 19 and *A. aegrifaciens* Kg 16. According to the data of denaturing PAGE, the MW of the subunit of all listed enzymes was ~31 kDa. According to gel filtration data, the MW of the phosphonatase was ~60–65 kDa, i.e., the enzymes in the native conformation were homodimers.

Both forms of *A. insolitus* Kg 19 phosphonatase (in the native and recombinant variants) had the same mobility during gel filtration (retention volume 255–260 mL), corresponding to an MW of 60–65 kDa. The preparations of PhnX19-RI and PhnX19-RII, preliminarily separated by affinity chromatography, upon subsequent application to an 8 × 80 mm DEAE-Toyopearl column were each eluted as one active peak at NaCl concentrations of 0.17 M and 0.3 M, respectively. An increase in the concentration of DTT in working buffers to 20 mM had no effect on the chromatographic separation of both native and recombinant forms of *A. insolitus* Kg 19 phosphonatase and did not cause the transition of one form into the other. MALDI-TOF analysis of trypsinolysis products of samples obtained by denaturing PAGE, both in a system without DTT and with a five-fold concentration, did not reveal significant differences in the peptide fingerprints of the two forms of the enzyme.

The presence of a polyhistidine tag did not significantly affect the characteristics of the enzymes. Parameters such as the range of optimal pH values, temperature optimum, and thermal stability were very similar for the native and recombinant phosphonatases.

Determination of the kinetic characteristics of *Achromobacter* phosphonatases showed that PhnX16 had a relatively low affinity to the substrate (PAA) and, respectively, low catalytic efficiency (*k*_cat_/*K*_m_ ratio) compared with PhnX19 phosphonatase (Table 2). Two forms of the enzyme from *A. insolitus* Kg 19 were, in turn, almost identical in their characteristics both in the native and recombinant forms and, in general, comparable in catalytic efficiency with *P. aeruginosa* phosphonatase, but significantly inferior to the wild-type *B. cereus* enzyme [19].

All phosphonatases under study featured substrate inhibition, expressed as a decrease of the observed enzyme activity at PAA concentrations of 3 mM and higher in the reaction mixture.

**Figure 4:**
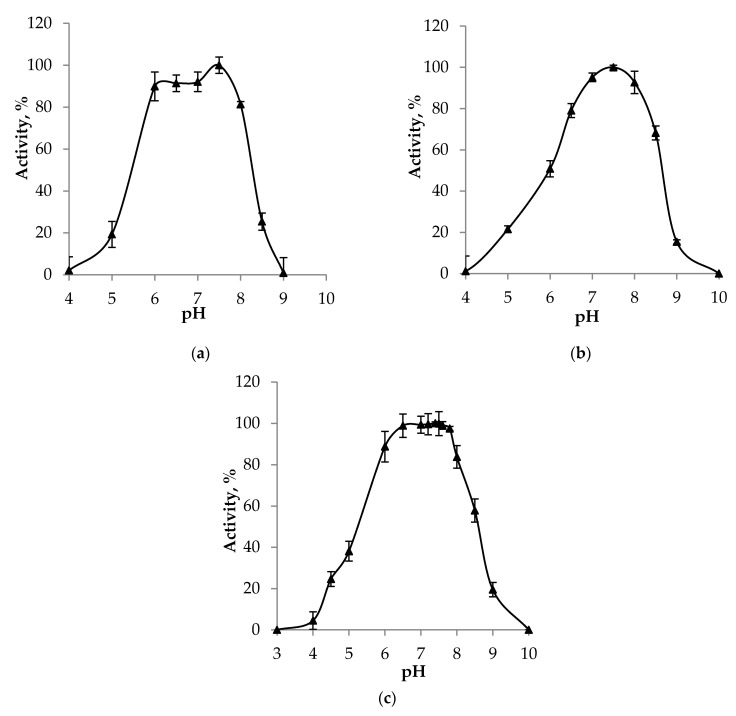
Dependence of the phosphonatase activity on the pH value of the medium. (**a**) PhnX16/16-R, (**b**) PhnX19-I/19-RI, and (**c**) PhnX19-II/19-RII. Vertical bars represent confidence intervals at a confidence level of 95%.

The enzymes preserved high activities within a relatively wide range of pH values, from 6.0 to 8.0, with an optimum of about 7.5 (Figure 4), though differences between the obtained pH profiles were statistically significant. 

**Figure 5:**
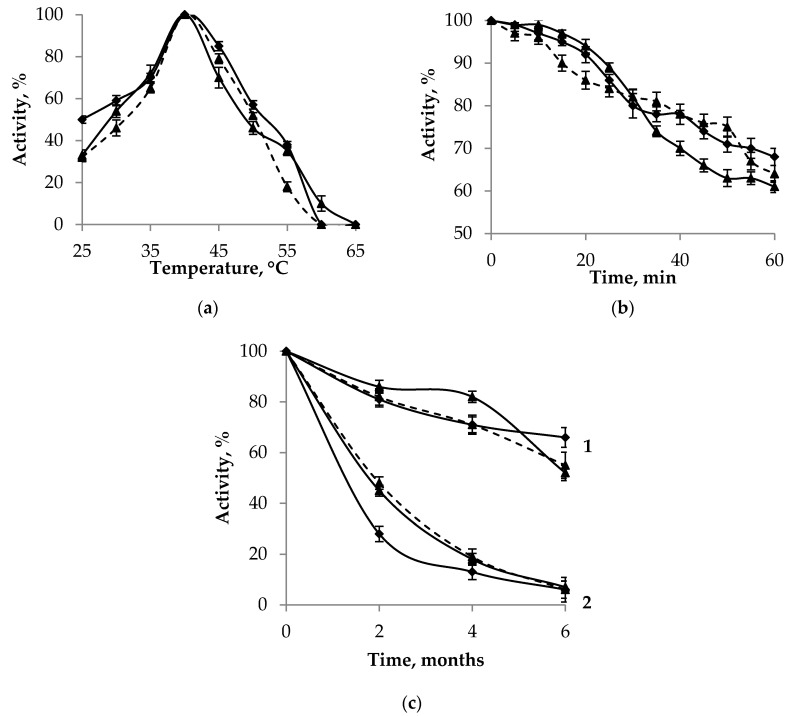
Phosphonatase activity at different temperatures and during storage. (**a**) Determination of the enzymes’ temperature optima. (**b**) Dynamics of phosphonatase inactivation at a temperature optimum (40 °C). (**c**) Decrease in phosphonatase activity during long-term storage at −20 °C (1) and +4 °C (2). Rhombi, PhnX16; triangles (solid lines), PhnX19-I; triangles (dashed line), PhnX19-II. Vertical bars represent confidence intervals at a confidence level of 95%.

The maximum activity of phosphonatases was observed at 40 °C (Figure 5a), and incubation at this temperature for 1 h led to 30% inactivation (Figure 5b). At temperatures higher than 60 °C, phosphonatases were completely inactivated. The purified enzyme preparations were relatively stable when stored frozen at −20 °C (Figure 5c) or −40° without any protectors or stabilizers: a decrease in activity over 6 months was 20–40% of the initial value. At +4 °C, up to 95% of phosphonatase activity was lost during the same period.

The results of the inhibitory analysis of native phosphonatases are shown in Table 3. Compounds such as GP, 2,4-DNPA, sodium borohydride, AMPA, 2-AEP, glutathione, and butylphosphonic acid (BPA) had both inhibitory and activating effects, i.e., were effectors [31]. Borohydride proved to be a competitive inhibitor in all cases, while 2,4-DNPA was uncompetitive for the PhnX19-I and PhnX19-II enzymes but, unexpectedly, had practically no effect on PhnX16. The enzyme from *A. aegrifaciens* Kg 16 was activated by AMPA and 2-AEP, while for *A. insolitus* Kg 19 phosphonatases, the latter (2-AEP) acted as an inhibitor. Glutathione increased the phosphonatase reaction rate in all cases, whereas GP acted as an uncompetitive inhibitor.

The activity of the investigated phosphonatases depended on the presence of metal ions in the reaction mixture. PAA hydrolysis was the most active in the presence of 10 mM Mg^2+^ (in the form of MgCl_2_). In the absence of magnesium, we observed trace activity of PhnX19 (both forms) and almost zero activity of PhnX16. Addition of 3 mM CuCl_2_ or 1 mM CoCl_2_ to the reaction mixture along with magnesium increased the observed reaction rate by 20–30% relative to the control. Ca^2+^, Ni^2+^, Fe^2+^, Mn^2+^, and Zn^2+^ ions had no noticeable effect on the phosphonatase activity.

The absorption spectra of pure preparations of all phosphonatases under study had no maxima characteristic of the known chromophore groups.

### 2.7. In Silico Study of the Structure and Functions of the Proteins

Prediction of the tertiary structures of PhnX16(16-R), PhnX19(19-R), and their subsequent comparison did not reveal significant factors that would unambiguously explain the differences in the properties and behavior of these proteins, primarily during column chromatography on anion exchange and affinity carriers. Superposition of the predicted three-dimensional structures onto the *B. cereus* phosphonatase model obtained by X-ray diffraction analysis [9] showed a high similarity of the models both in terms of the structure of hydrophobic sites of subunit binding and the formation of active centers by cap and core domains [32] (Figure S6). 

According to the results of primary sequences’ alignment, out of 12 amino acid residues forming the active center of *B. cereus* phosphonatase [33], 9 residues were identical to those of PhnX16/PhnX19, including those directly involved in the catalysis of Asp12, Lys53, and His56 (Asp16, Lys57, and His60 for achromobacteria). There were also three substitutions (Cys22Ser, Thr126Ser, and Gly185Asp) (Figure S7). Previously, it has been shown that the introduction of the first or last of these substitutions into the structure of *B. cereus* phosphonatase by directed mutagenesis led to a decrease in the activity of the enzyme relative to the wild-type [9,34].

No possibility of forming intramolecular disulfide bonds in the predicted native conformations was revealed for all phosphonatases under study.

No potential N- or O-glycosylation sites in phosphonatases were found. A search for possible phosphorylation sites revealed the presence of five identical sites for PhnX19 and PhnX16 (residues Ser4, Ser130, Ser135, Ser180, and Ser268) and one unique site for PhnX19—Ser146.

There were no signs of the presence of signal sequences in all investigated variants of the PhnX, HpnW, and PhnHD proteins, which could indicate their extracellular localization.

Analysis of conserved segments of the polypeptide chain of putative oxidoreductase HpnW procured neither the unambiguous identification of the function of the enzyme nor the detection of its close homologs. According to CDD data, the TIGR03364 clade, to which HpnW belongs, is related to D-amino acid oxidases, and the genomic context of HpnW-like proteins often includes genes for OP catabolism. Prediction of the three-dimensional structure and search for possible ligands confirmed a high probability of the presence of a non-covalently bound FAD coenzyme in the protein globule. Possible substrates capable of binding in the predicted HpnW active center in the presence of FAD include glycolic acid, glycerol-3-phosphate, 2-aminobenzoic acid, N,N-dimethylglycine, and acetylaminoacetic acid.

**Figure 6:**
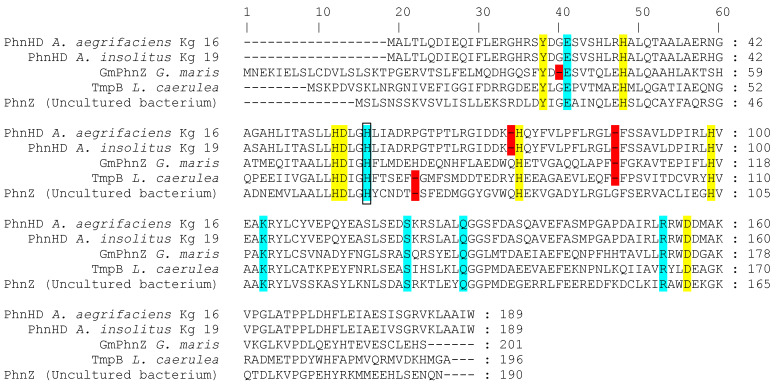
Alignment of the amino acid sequences of the known and putative metal-containing OP dioxygenases. Blue, residues observed to bind substrates in TmpB and *Gm*PhnZ1. Yellow, residues observed to bind the diiron cofactor in TmpB and PhnZ. Red, gaps. The frame highlights the fifth histidine invariant for the TIGR03276 clade.

Putative PhnHD proteins of the investigated bacteria belong, according to CDD data, to the small clade TIGR03276 within the superfamily of metal-containing phosphohydrolases, HDc. Representatives of this clade are distinguished by five invariant histidine residues, and their genomic context often includes genes of a number of OP catabolism proteins, which corresponds to the location of the PhnHD genes in close proximity to phosphonatase operons in the genomes of the achromobacteria. Initially, PhnHD of *A. insolitus* Kg 19 and *A. aegrifaciens* Kg 16 were annotated as phosphohydrolases; later, a high degree of homology was noted with the iron-containing HAEP dioxygenase of marine prokaryotes PhnZ (BLAST score = 95; amino acid composition identity 30%), HMPA dioxygenase *Gm*PhnZ1 from *G. maris* (BLAST score = 114; identity 34%), and TmpB (BLAST score = 100; identity 33%). Alignment of the amino acid sequences of the aforementioned proteins (Figure 6) showed the presence of all conserved residues that had been known [12,13,35] to bind the diiron cofactor (Figure 6, yellow) and OP substrate (Figure 6, cyan) in relatively similar positions, despite several gaps (Figure 6, red), as well as the fifth conserved histidine as a distinctive feature of the TIGR03276 clade.

The predicted three-dimensional PhnHD structure of *A. insolitus* Kg 19 and *A. aegrifaciens* Kg 16 was quite similar to the corresponding TmpB structure determined by X-ray diffraction analysis [12], especially in the region of the assumed active center. Amino acid residues involved in the oxidative rupture of the C–P bond of the TmpB substrate, according to modeling data, formed a cavity in PhnHD, providing the binding of two iron ions and a molecule of one of the possible substrates (Figure S8): either citrate anion, or 2,3,4,5,6-pentahydroxycyclohexanone, or else HAEP, out of which only the latter could be presumed as possible with all considerations taken into account.

## 3. Discussion

To date, the structure and function of phosphonatases have been comprehensively studied in *B. cereus* [6,9,19,34] and, in less detail, in *P. putida* and *Klebsiella aerogenes* [36], *S. typhimurium* [8], and *P. aeruginosa* [37]. The phosphonatase operons of these bacteria are induced either by P_i_ deficiency or in the presence of 2-AEP and perform the function of catabolism of the latter. The recent finding of PbfA lyase and PbfB, PbfC, and PbfD oxidoreductases [10,14] has shown that the PhnW–PhnX phosphonatase pathway can in many cases be supplemented with novel enzymes and thus provide for utilization of compounds other than 2-AEP and PAA (Figure 1, *5*, *9*). In the present work, it was confirmed that the phosphonatase pathway still cannot be considered fully investigated, and its variants that are distinct from those described for *B. cereus* are present not only in marine bacteria, but in soil microbiota as well.

The impact of a phosphorus source in the medium on phosphonatase activity in cell homogenates (Figure 2) did not fully correspond to known models of enzyme expression regulation. The yield of the enzyme decreased or increased several times, respectively, during cultivation on P_i_ or in the absence of a phosphorus source, but also depended on the presence of a particular OP in the medium. This manifested differently in the studied achromobacteria. Most notable was a high activity of phosphonatase during the growth on GP, comparable to the activity during the growth on 2-AEP for *A. aegrifaciens* and significantly exceeding that in *A. insolitus*. These data correlate with the presence of a LysR transcription activator gene in the immediate vicinity of the phosphonatase operon (Figure 3). The protein regulators (NGT17081.1 and MDR7944340.1) of the investigated bacteria differed in eight amino acid residues. Due to the wide diversity of LysR proteins, it is currently impossible to relate these differences to the dynamics of LysR’s interaction with its ligands. Non-specific binding of LysR to the phosphonic group seems unlikely, since MPA did not increase the phosphonatase yield, similar to earlier data for *O. anthropi* [24]. At the same time, dependence of the enzyme activity on exogenous P_i_ suggests multifactor regulation of phosphonatase expression similar to *K. aerogenes* IFO 12010 [36], *S. typhimurium* [25,27], or *P. aeruginosa* [37], which should involve simultaneous action of a substrate-specific transcriptional regulator alongside the PhoB/PhoR system. We found no sequences corresponding to known *pho* box variants in the genome context of phosphonatase operons or *lysR* and *phnHD* genes in achromobacteria, so the exact mechanism of P_i_-dependent regulation of the expression of the abovementioned genes was not obvious. The following two-stage scheme appears logical: a decrease in the concentration of exogenous P_i_ activates the expression of LysR, which binds with the ligand (substrate of the phosphonatase pathway) and increases the rate of transcription of *hpnWphnSXUT* genes. The ability of GP to bind to LysR and thus induce phosphonatase synthesis seems plausible according to existing data but would require further investigation.

Although the proposed schemes for native phosphonatases’ isolation provided protein preparations with a sufficient degree of purity, the total enzyme yields were very low even after the cultivation of wild-type strains on media with GP, which seems to be typical of OP catabolism enzymes due to the relatively low demand for phosphorus compared with carbon [24]. Taking into account the almost identical characteristics of the native and recombinant enzymes, the heterologous expression of PhnX16R and PhnX19R in *E. coli* was a more optimal method for studying *Achromobacter* phosphonatases.

The phenomenon of phosphonatases separating into two active fractions in column chromatography has not been previously described in the literature. It can be asserted that in *A. insolitus*, this was not the result of the presence of an isoenzyme with phosphonatase activity or the separation of the dimeric form of native PhnX19 into subunits. It did not occur, either, due to the formation of intra- or inter-molecular disulfide bonds. Post-translational modifications in the form of glycosylation or phosphorylation, or to the binding of PhnX19 to a certain ligand specific to strain Kg 19, seem improbable.

For *B. cereus* phosphonatase, a dynamic equilibrium between “open” and “closed” conformational states has been described [19,32]. The assumption that PhnX19-I or PhnX19-II is one of similar conformers in a metastable state seems unfounded, as the catalytic properties of the “stabilized” form of the enzyme must be severely impaired [33], which was not the observed (Table 2). This also does not explain the discrepancies in the binding of the two phosphonatase forms on affine or anion-exchange resins, as the number of aspartate and glutamate residues in contact with the solution differed by only 7% for closed and open conformations, and even less so for histidine residues.

The phosphonatases under study, in general, were significantly inferior in catalytic efficiency to the wild-type *B. cereus* enzyme, and the characteristics of *A. insolitus* Kg 19 proteins were comparable to those of *P. aeruginosa* (Table 2). Changes in the composition of amino acids involved in the formation of the active phosphonatase center and the binding of the Mg^2+^ cofactor often led to a decrease or disappearance of the enzyme activity. Similar to mutant variants of *B. cereus* PhnX with a significantly reduced enzyme reaction rates, three amino acid substitutions—Cys22Ser [9], Gly185Asp, and Asp190Gly [34]—were found in respective positions in both *A. insolitus* Kg 19 and *A. aegrifaciens* Kg 16 (Figure S7). This would explain the worst characteristics of the enzymes under study but raises the question of the evolutionary prerequisites for the fixation of relatively ineffective variants of a protein important for maintaining phosphorus homeostasis.

Short-term incubation of PhnX16 in the presence of 2 mM 2,4-DNPA did not lead to a significant decrease in enzyme activity, unlike PhnX19 (Table 3). A similar behavior has been described for the closed conformation of *B. cereus* phosphonatase stabilized by an inert substrate, which provided protection of the Lys53 residue from the action of an inhibitor [19]. A shift in equilibrium toward an open or closed conformation should negatively affect the activity of the enzyme [33], which is consistent with the relatively low catalytic efficiency of PhnX16/16-R relative to PhnX19/19-R (Table 2).

The temperature optima of *Achromobacter* phosphonatases and their stability during storage corresponded to the literature data overall, except for a better tolerance of the investigated proteins to multiple freezing compared with the *P. aeruginosa* enzyme [37]. The range of optimal pH values, which in phosphonatase is determined by the conserved amino acid residues of aspartate, lysine, and histidine in the active center, was expectedly located in the near-neutral region (Figure 4).

While the studied enzymes did not prove to be fundamentally different from known phosphonatases, they demonstrated certain peculiar traits. However, further deliberation on the nature of the differences between PhnX19-I, PhnX16, and PhnX19-II will be speculative without experimental determination of the structures of the abovementioned variants of phosphonatase, which was beyond the scope of this work.

The composition of gene clusters that included the phosphonatase operon and functionally related genes in *A. insolitus* Kg 19 and *A. aegrifaciens* Kg 16 were unexpected, primarily with regard to the location of the 2-AEP:pyruvate transaminase gene *phnW* in another part of the chromosome, separately from any operon associated with the utilization of OP, and the presence, instead, of a putative flavin oxidoreductase gene, *hpnW*. Here, we observed a similar composition of the phosphonatase gene cluster in the GP degrader *O. anthropi* GPK 3, though in contrast to achromobacteria, its genome completely lacked any homologs of *phnW* (data are currently being prepared for publication).

Among the predicted in silico HpnW substrates, acetylaminoacetate and dimethylglycine both contained secondary or tertiary amines as a part of nitrogen–carbon–carboxyl moieties, similar to those of GP or *N*-acetylglyphosate. This conforms well to recent information that several types of novel flavin oxidoreductases attack the bond between secondary amine and ethylphosphonic moieties, thus facilitating MAEP catabolism through the phosphonatase pathway [14]. While it is plausible that HpnWs of the studied achromobacteria have similar functions, the question of the possible specificity of these enzymes toward GP is especially interesting in light of, firstly, the increased yields of phosphonatase in the presence of the herbicide and, secondly, the absence of GP oxidoreductase homologs in these bacteria, along with the ability to form AMPA when grown on GP [28]. As the PhnW substrate was an activator of expression of some LysR-regulated phosphonatase operons [27], one could assume that, at the substitution of PhnW with HpnW, it is the substrate of the latter or a structurally close compound that will act as the phosphonatase inducer.

Discussion of the possible function of the putative protein PhnHD, automatically annotated as phosphohydrolase, requires caution before experimental data are obtained. As the Fe^2+^/Fe^3+^-containing dioxygenase TmpB has also been initially annotated as a phosphohydrolase of the HDc superfamily, the H...HD...H...H...D motif, binding two metal ions rather than one (as is the case with hydrolases), has been proposed as an identifier of bimetallic oxygenases [12]. While this feature is not absolutely reliable, as some unusual bimetallic phosphohydrolases with this motif are known [35], the presence of the fifth conserved histidine characteristic of the TIGR03276 clade (Figure 6, frame) is a stronger indication of the involvement of putative proteins in the metabolism of OP as dioxygenases. Among the predicted substrates that correspond to the assumed configuration of the PhnHD active center (Figure S8), HAEP stands out, also being the common ligand for three known C–P bond-cleaving dioxygenases. Still, *Gm*PhnZ and TmpB are much more specific toward their natural substrates (HMPA and HTAEP, respectively) [12,13] and transform HAEP inefficiently, despite comprising a similar set of amino acid residues that bind the substrate (Figure 6, cyan) and diiron cofactor (Figure 6, yellow). There are differences, though, in the relative positioning of conserved residues, depicted as gaps in primary structures’ alignment (Figure 6, red), which could lead to distinct configurations of active centers and explain discrepancies in the substrate specificity. In this regard, PhnHDs are not identical to either of the abovementioned dioxygenases.

The enzymes that catabolize HAEP and similar compounds have so far been characterized in detail only in marine microorganisms [2,10,12,35], where oxidative degradation of OP was an alternative to the absent phosphonatase pathway. This would be redundant both for the achromobacteria under study and for some pseudomonads that also possess TIGR03276 clade proteins, along with PhnW and PhnX, but lack PhnY* [2,35]. In the case of *A. insolitus* Kg 19 and *A. aegrifaciens* Kg 16, the structure of the phosphonatase gene cluster is even more unusual compared to pseudomonads due to the substitution of PhnW transaminase with putative HpnW oxidoreductase, which can either be involved in the formation of a substrate for phosphonatase, similar to Pbf enzymes [14], or serve as a substrate source for PhnHD.

The answer to the conundrum of the functions of HpnW and PhnHD is of great interest and would provide better understanding of the role of the phosphonatase gene cluster in the catabolism of OP, including GP and structurally similar compounds. This will be a priority task for further research in this field.

## 4. Materials and Methods

Analytical- or higher-grade reagents were used. All operations with commercial sets of reagents were carried out in accordance with the manufacturers’ instructions unless indicated otherwise.

### 4.1. Bacterial Cultures

OP-degrading bacteria were grown on liquid and agar mineral media MS1, with GP, AMPA, MPA, 2-AEP or P_i_ as the sole sources of phosphorus, with preliminary 48 h phosphorus starvation. The compounds were introduced in terms of the final total phosphorus concentration of 0.92 mg per liter of cultivation medium [28,38]. *E. coli* cultures were grown on liquid and agar Luria–Bertani (LB) media.

Periodic cultivation of bacteria to produce biomass for subsequent isolation of proteins was carried out in 200 mL of liquid media in 750 mL shake flasks at 28 °C and 220 rpm for *Achromobacter* and at 37 °C and 160 rpm for *E. coli.*

The growth of cultures was controlled by measuring the optical density on a UVmini-1240 spectrophotometer (Shimadzu, Kyoto, Japan) at a wavelength of 560 nm (OD_560_). Biomass was calculated using the empirical formula *M* = 0.5 × OD_560_, where *M* is dry biomass, in g L^−1^ [39].

### 4.2. Taxonomic Position of Bacteria under Study

Bacteria of the genus *Achromobacter* were isolated by the enrichment culture method from samples of agricultural soils in the Krasnodar Territory and treated for a long time with the Roundup herbicide [38,40].

The species identity of the GP-degrading strains was determined by analyzing the nucleotide reductase gene *nrdA* [41,42], as described earlier [28].

### 4.3. Whole-Genome Sequencing and Analysis of Genomic Data

Sample preparation and whole-genome sequencing of the bacterial strains chosen for this work have been described previously [28,40].

The enzyme systems cleaving the C–P bond were identified by revealing the sequences homologous to the marker genes of the key enzyme systems, *phnJ* (C–P lyase) and *phnX*, followed by a study of the genomic context. Additionally, we searched for the homologs of the genes *phnW*, *gox* (GP oxidoreductase) [43] (GU214711.1, https://www.ncbi.nlm.nih.gov/nuccore/GU214711.1, accessed on 25 October 2023), and *gltA* (GP-acetyltransferase) [44] (AY597418.1, https://www.ncbi.nlm.nih.gov/nuccore/AY597418, accessed on 25 October 2023), and other genes that determine the ability of a cell for OP catabolism.

### 4.4. In Silico Analysis of the Proteins

Prediction of the three-dimensional structures of the investigated proteins and their active centers was carried out using the IntFOLD toolkit version 7.0 (https://www.reading.ac.uk/bioinf/index.html, accessed on 27 October 2023) [45,46]. To determine possible ligands and substrates in the absence of experimental data, we analyzed the three best models for each protein under study (with the *p*-criterion value of 10^−11^ and lower, and an overall model quality value of no less than 0.62) using the FunFOLDQA tool within the IntFOLD system. The resulting three-dimensional structures were analyzed by the 3D-Mol Viewer (Invitrogen, Carlsbad, CA, USA), Geneious Pro 4.8.5 (Biomatters, Auckland, New Zealand), and NCBI iCn3D tools [47]. The surface of the protein globule in contact with the solution was calculated using the 3D-Mol Viewer with Varshney’s method.

The amino acid sequences were aligned in the Geneious Pro 4.8.5 program (Geneious method, global alignment with free end gaps).

The superposition and alignment of the three-dimensional protein structures was carried out with Softberry 3D-Match (http://www.softberry.com/berry.phtml?topic=3dmatch&group=programs&subgroup=3d-expl, accessed on 5 November 2023).

The SignalP 6.0 service was used to predict the presence of a signal peptide in the protein sequence of the investigated proteins and the location of the proteolytic cleavage site [48].

The homology of putative proteins, revealed by the analysis of *Achromobacter* genomes, to known protein families was studied using the NCBI Conserved Domain Database [49].

The search for potential glycosylation sites was carried out using the Glycopp v1.0 service [50] (https://webs.iiitd.edu.in/raghava/glycopp/, accessed on 4 November 2023). For N-glycosylation, use was made of the BPP method, SVM threshold = 0; while for O-glycosylation, the ASA + PPP method was used, SVM threshold = 0.

The search for potential phosphorylation sites by serine and threonine residues was performed by the NetPhosBac 1.0 tool [51] (https://services.healthtech.dtu.dk/services/NetPhosBac-1.0/, accessed on 5 November 2023).

### 4.5. Cloning of Phosphonatase Genes into Expression Vectors and Construction of Super-Producers

The genomic DNA of *A. insolitus* Kg 19 and *A. aegrifaciens* Kg 16 was isolated with the DiaGene 3318 kit (Dia-M, Moscow, Russia) and used as a matrix for the PCR with KOD Hot Start polymerase (Merck KGaA, Darmstadt, Germany), with primers 5′-TAACATATGACTGTTTCGCCTTTGCC-3′ (forward), 5′-TTCTCGAGGGGACGCTGGCC-3′ (reverse for strain Kg 19), or 5′-TTCTCGAGGGGGCGCTGGCC-3′ (reverse for strain Kg 16). The PCR products were purified using the ZymoGene DNA Clean and Concentrator kit (ZymoResearch, Irvine, CA, USA) and cloned into the pET-22b vector (Merck) at NdeI/XhoI recognition sites using NdeI and XhoI FastDigest restrictases and T4 DNA ligase (Thermo Fisher Scientific, Waltham, MA, USA). The ligase mixture was used to transform chemically competent *E. coli* DH5α cells. Transformed cells were selected on an agar LB medium with 100 mg l^−1^ of ampicillin. Cells from individual colonies were seeded onto LB liquid media with ampicillin, grown to OD_560_ ≈ 4.5, and precipitated at 10,000× *g* for 5 min. Plasmid DNA was isolated using the DiaGene 3316 kit (Dia-M, Moscow, Russia). The presence of an insert was checked by treatment with NdeI and XhoI FastDigest restrictases, followed by electrophoresis in 1% agarose gel with ethidium bromide. The structures containing the expected insert were used to transform chemically competent *E. coli* BL21 (DE3) cells to obtain phosphonatase super-producers.

### 4.6. Production of Cell-Free Extracts

We used cultures at the stage of growth retardation or immediately after phosphorus starvation for subsequent enzyme measurements and purification [24]. Culture fluids were centrifuged at 4200× *g* for 30 min, the precipitate was resuspended in 300 mL of a working buffer (50 mM Tris-HCl, pH 7.5, 10 mM MgCl_2_, and 0.1 mM DTT), and re-centrifuged under the same conditions. Then, the precipitate was resuspended in 15 mL of a working buffer, frozen at −40 °C, and extruded in a frozen state at the IBPM press installation (Institute for Biological Instrumentation RAS, Pushchino, Russia) at an operating pressure of 0.9 MPa.

Then, 100 units of DNase I from bovine pancreas (Sigma, St. Louis, MO, USA) and 15 mL of working buffer were added to the resulting homogenate and centrifuged at 25,000× *g* for 75 min at 4 °C. The supernatant liquid was passed through Minisart membrane filters (Sartorius, Goettingen, Germany) with a pore diameter of 0.2 µm, and the filtrate was stored in ice.

To produce cell-free extracts of recombinant phosphonatase producers, *E. coli* BL21 (DE3) cultures were inoculated in 5 mL of an LB medium with ampicillin (100 mg/l), grown to OD_560_ ≈ 4.5, and used for inoculation of shake flasks with 200 mL of the LB medium with ampicillin each. The cultures were grown to OD_560_ ≈ 0.5 at 37 °C. Isopropyl-β-D-1-thiogalactopyranoside (IPTG) was added to a final concentration of 1 mM, then the contents were cultivated for 15 h at 20 °C. The culture fluids were centrifuged at 7800× *g* for 30 min, and the precipitate was resuspended in 200 mL of a working buffer and re-centrifuged at 3000× *g* for 30 min. The precipitate was resuspended in 20 mL of a working buffer on ice and homogenized on a Soniprep 150 Plus ultrasonic disintegrator (MSE, Heathfield, UK) at a 50% amplitude by sonicating 5 times for 1 min, with 1 min breaks in an ice bath. Then, 100 units of DNase I from bovine pancreas were added to the homogenate, and the contents were then centrifuged at 25,000× *g* for 75 min at 4 °C. The supernatant was passed through Minisart membrane filters with a pore diameter of 0.2 µm and stored at ×40 °C.

### 4.7. Measurement of Enzyme Activity

The hydrolytic activity of phosphonatase against PAA was measured in a conjugate reaction with NADH and alcohol dehydrogenase (ADH) in working buffer [24]. The amount of the enzyme hydrolyzing 1 µmol of PAA per minute was taken for one unit of activity (U). PAA was synthesized immediately before the measurements [52]. The resulting PAA solution was aliquoted at 100 µL and stored at −70 °C using a fresh aliquot for each series of measurements. The test cuvettes with phosphonatase, PAA, NADH, and ADH were measured against control cuvettes with a similar composition but without phosphonatase to eliminate the non-specific decrease of absorption at 340 nm due to trace amounts of acetaldehyde in PAA. Measurements of reaction mixtures with NADH, phosphonatase, and PAA, or NADH, PAA, and ADH against buffer, were used as a negative control. All measurements were carried out at 25 °C unless indicated otherwise.

### 4.8. Isolation of Native Phosphonatases

Phosphonatases of wild-type strains were purified by column chromatography at an ÄKTA Pure facility (GE Healthcare, Chicago, IL, USA) in a working buffer. A cell-free extract was passed through a 0.2-µm MiniSart filter and applied onto a 16 × 700 mm column with the DEAE-Toyopearl anion exchange carrier (TOSOH, Tokyo, Japan). Elution was carried out with a linear NaCl gradient within the range of 0–2.0 M. Fractions containing phosphonatase activity were concentrated using Vivaspin 20 membrane concentrators (Sartorius, Goettingen, Germany), after which gel filtration was performed on a HiLoad Superdex 200 column, 26 × 600 mm (GE Healthcare), pre-calibrated with protein standards. An active fraction corresponding to a molecular weight (MW) of ~65 kDa was concentrated, then applied to an 8 × 80 mm column with the DEAE-Toyopearl carrier and eluted with a linear NaCl gradient in a concentration range of 0–0.6 M. Phosphonatase preparations obtained after repeated anion-exchange chromatography were homogeneous according to denaturing electrophoresis data and were used for further studies of the enzyme.

### 4.9. Isolation of Recombinant Phosphonatases

To obtain pure preparations of recombinant phosphonatases bearing polyhistidine tags at C-ends, we performed column chromatography on a HisTrap FF affinity column of a 5 mL volume (GE Healthcare, Chicago, IL, USA) in a working buffer with imidazole. A cell-free extract pre-dialyzed against a working buffer with 20 mM imidazole was applied to the column, and proteins were eluted with a stepwise imidazole gradient with concentrations of 20, 65, 110, and 300 mM, with each step the length of 3 column volumes. Active fractions containing electrophoretically homogeneous phosphonatase preparations were dialyzed against a working buffer without imidazole and used for further work.

Pure preparations of native and recombinant enzymes were stored in a working buffer at −40 °C.

### 4.10. Electrophoresis of Proteins in Polyacrylamide Gel

Denaturing disk electrophoresis (SDS-PAGE) was performed in 12% polyacrylamide gel with 0.2% sodium dodecyl sulfate by the Laemmli method using a Mini-Protean device (Bio-Rad, Hercules, CA, USA). The PAGE mode was 120 V, 30 mA, 30 W, and current stabilization. Before application, protein preparations were mixed with two volumes of a freshly prepared sample buffer with 100 mM dithiothreitol (DTT) and boiled in a water bath for 10 min. Protein staining in polyacrylamide gel was carried out either with the Blue R reagent (Serva, Heidelberg, Germany) or, at low protein concentrations in the samples, using a Silver Stain Plus Kit (Bio-Rad), in accordance with the manufacturer’s instructions.

### 4.11. MALDI-TOF Assay

After denaturing PAGE, the band corresponding to the investigated protein was excised from the gel, washed twice with acetonitrile, and then treated with trypsin (Promega, Madison, WI, USA). The mass spectra of the obtained proteins were recorded using an Ultraflex MALDI-TOF/TOF mass spectrometer (Bruker Daltonics, Bremen, Germany). The accuracy of measuring the masses of fragment ions was within 1 Da. Proteins were identified in the MASCOT program (www.matrixscience.com, accessed 13 January 2021). The NCBI database without restrictions for taxa was used for the search. Candidate proteins were considered reliably identified at a score greater than 83 (*p* < 0.05). The search for homologs of the isolated proteins was carried out using the NCBI–Protein BLAST database (https://blast.ncbi.nlm.nih.gov, accessed 13 January 2021).

### 4.12. Characterization of Native and Recombinant Phosphonatases

1. The molecular weights of denatured proteins were determined based on the data of SDS-PAGE using the calibration curve relating the electrophoretic mobility of protein standards (Unstained Protein Molecular Weight Markers, Thermo Fisher Scientific, Waltham, MA, USA) to the logarithm of their molecular weights.
2. The molecular weights of native proteins were assessed using gel filtration on a HiLoad Superdex 200 column, 26 × 600 mm, based on a calibration curve expressing the dependence between the logarithm of the molecular weight of the proteins (from the Gel Filtration Molecular Weight Markers Kit for Molecular Weights 12,000–200,000 Da (Sigma, St. Louis, MO, USA)) and the corresponding volume of elution.
3. The phosphonatase activity of A. insolitus Kg 19 was separated into two active fractions by conducting:
  - Gel filtration of the preparations of two forms of the enzyme in both native and recombinant forms on a HiLoad Superdex 200 column, 26 × 600 mm, in a working buffer with a DTT concentration of 20 mM.
  - Ion-exchange chromatography of recombinant phosphonatase forms on an 8 × 80 mm column with the DEAE-Toyopearl carrier in a linear NaCl gradient in a concentration range from 0 to 2 M.
  - Denaturing PAGE using a buffer for samples with a five-fold (500 mM) concentration of DTT and without DTT, followed by MALDI-TOF analysis.
4. The optimal pH value was determined using a universal buffer [53] with 10 mM MgCl_2_ and 0.1 mM DTT. The activity was measured in the pH range from 4 to 10. The effect of universal buffer components on phosphonatase activity was evaluated by measuring the enzyme activity in a working buffer with 40 mM boric acid or 40 mM phosphoric acid.
5. The determination of temperature optima was carried out in a working buffer within the temperature range of 25–65 °C using a UV-1800 spectrophotometer (Shimadzu, Kyoto, Japan) with thermostatted cells. Cuvettes with reaction mixtures were incubated in cells for 3 min immediately before a measurement. Enzyme preparations before their introduction into a cuvette were incubated for 1 min in a water bath of appropriate temperature. To assess their thermal stability, phosphonatase preparations were incubated at 22 °C, 45 °C, and 65 °C for 1 h, measuring the residual activity every 10 min.
6. The stability of phosphonatases during long-term storage was studied for 6 months, with activity measurements every 2 months, incubating enzymes at 22 °C, 4 °C, −20 °C, and −40 °C.
7. The kinetic constants were determined based on the empirically established correspondence of the dependence of the phosphonatase reaction rate on the concentration of the Michaelis model. The linearization of the kinetic curve was performed in Hanes–Woolf coordinates ([*S*]/[*S*] · *V*_0_^−1^, where [*S*] is the concentration of the substrate, in mM, and *V*_0_ is the initial reaction rate, in mmol min^−1^). The value modulo [*S*] at the point of intersection of the graph with the abscissa axis was taken to be *K*_m_, and the ratio of 1 to the tangent of the angle of inclination of the graph to be *V*_max_.
8. The phosphonatases’ optical absorption spectra were recorded on a UV-1800 spectrophotometer in the wavelength range of 200–900 nm in quartz cuvettes with an optical path length of 1 cm.
9. The effect of metal ions on phosphonatase activity was studied by determining the activity of enzyme preparations in a working buffer: (a) with 10 mM MgCl_2_ (positive control), (b) without metals (negative control), and (c) with one of the salts: MgCl_2_ × 6H_2_O, NiCl_2_ × 6H_2_O, CoCl_2_ × 6H_2_O, MnCl_2_ × 4H_2_O, CuCl_2_, FeCl_2_, or ZnSO_4_, in concentrations of 1–40 mM (experiment).
10. Inhibitory analysis of phosphonatases was performed using the following compounds as possible effectors: GP, 2-AEP, AMPA, glutathione, sodium borohydride, 2,4-dinitrophenyl acetate (2,4-DNPA), and butyl phosphonate. Measurements were carried out within the range of effector concentrations of 0–40 mM and substrate concentrations of 0.1–5 mM. The type of inhibition and the inhibition constant were determined by the vector method [31].

### 4.13. Statistical Processing of the Results

All experiments with living bacterial cultures were performed in three repeats, and measurements of enzymatic activity in five repeats. For each sample, the standard deviation and the confidence interval for the normal distribution were calculated at a confidence level of 95%. Additionally, single- or two-factor ANOVA was performed for all datasets involving enzyme assays, and *p*-values were tested against a significance level of 0.05.

## 5. Conclusions

The data obtained in this work showed the presence of a functioning phosphonatase pathway in highly effective OP soil degraders of the genus *Achomobacter*, the synthesis of a key enzyme of which (PhnX) was promoted not only during the growth on a natural substrate, 2-AEP, but also on a synthetic herbicide, GP. Purified preparations of native and recombinant *Achomobacter* phosphonatases demonstrated lower catalytic efficiency, as compared with the *B. cereus* enzyme, which was nevertheless sufficient to supply the cell with orthophosphate when grown on 2-AEP. The unusual properties of the investigated phosphonatases, namely the relative resistance of the *A. aegrifaciens* Kg 16 enzyme to the action of the 2,4-DNPA inhibitor and the presence of two forms of the protein close by their properties in *A. insolitus* Kg 19, were evidently not related to covalent modifications of the protein molecules and require an in-depth study of the tertiary and quaternary structures of the proteins to explain.

The increased phosphonatase activity during the growth on a medium with GP may be related to the substitution of *phnW* cistron (transaminase) in *Achomobacter* phosphonatase operons with *hpnW* cistron (flavin oxidoreductase). The predicted substrates of the latter include compounds structurally similar to GP, which, along with 2-AEP, may form active complexes with the LysR transcription regulator, the gene of which was found in a single cluster with the phosphonatase operon and the gene of the putative bimetallic oxygenase, PhnHD. The role of the latter is not fully understood, and the spectrum of substrates may include 2-AEP, HAEP, or HTAEP.

## Figures and Tables

**Table 1:** Maximum biomass of bacteria grown on media with different sources of phosphorus.

Microorganism (VKM Collection Number)	Dry Biomass, g/L Cultivation Medium				
	GP	MPA	2-AEP	P_i_	Without Phosphorus
*A. insolitus* Kg 19 (B-3295)	1.6 ± 0.2	2.4 ± 0.2	2.7 ± 0.1	2.9 ± 0.2	0.2 ± 0.04
*A. insolitus* Kg 13 (B-3294)	2.1 ± 0.4	2.6 ± 0.3	3.0 ± 0.2	3.1 ± 0.3	0.3 ± 0.05
*A. aegrifaciens* Kg 16 (B-2534D)	1.2 ± 0.1	2.3 ± 0.2	1.7 ± 0.2	2.8 ± 0.1	0.2 ± 0.04
*A. aegrifaciens* Km 11A (B-3296)	0.7 ± 0.2	1.9 ± 0.1	2.3 ± 0.1	2.9 ± 0.1	0.2 ± 0.03

**Table 2:** Kinetic characteristics of the phosphonatases under study, as compared with the literature enzymes from *P. aeruginosa* [30] and *B. cereus* [19].

Enzyme	*K*_m_, μM	*V*_max_, U mg^−1^ Protein	*k*_cat_, s^−1^	*k*_cat_/*K*_m_, l mol^−1^ s^−1^
*P. aeruginosa* PhnX	200	10.0	5	2.5 × 10^4^
Wild-type *B. cereus* PhnX	33 ± 2	29.7 ± 2	15 ± 1	4.6 × 10^5^
*B. cereus* C22S * PhnX	33 ± 5	4.6 ± 0.18	2.26 ± 0.09	6.9 × 10^4^
*B. cereus* PhnX G185D/D190G *	54 ± 3	3.4 ± 0.6	1.7 ± 0.3	3.1 × 10^4^
PhnX16	889 ± 138	1.20 ± 0.2	0.6 ± 0.1	6.7 × 10^2^
PhnX16R	641 ± 23	1.12 ± 0.1	0.56 ± 0.05	8.75 × 10^2^
PhnX19-I	153 ± 21	2.5 ± 0.2	1.3 ± 0.1	8.7 × 10^3^
PhnX19-II	171 ± 26	2.3 ± 0.3	1.2 ± 0.15	7.1 × 10^3^
PhnX19-RI	150 ± 7	3.5 ± 0.1	1.8 ± 0.05	1.2 × 10^4^
PhnX19-RII	162 ± 9	3.2 ± 0.16	1.6 ± 0.08	1.0 × 10^4^

* Mutant strains.

**Table 3:** Effectors of *A. aegrifaciens* Kg 16 and *A. insolitus* Kg 19 phosphonatases.

Enzyme	Parameter	Effectors						
		GP	2,4-DNPA	NaBH_4_	AMPA	2-AEP	Glutathione	BPA
PhnX16	Effect	INH	nd	INH	AC	AC	AC	INH
	Type	uncomp		comp	comp	comp	noncomp	noncomp
	*K*_i_ (*K*_a_)	11.60		1.85	43.5	21	4.34	0.75
PhnX19-I	Effect	INH	INH	INH	nd	INH	AC	INH
	Type	uncomp	noncomp	comp		comp	noncomp	noncomp
	*K*_i_ (*K*_a_)	4.0	0.73	1.50		2.2	4.44	0.39
PhnX19-II	Effect	INH	INH	INH	nd	INH	AC	INH
	Type	uncomp	noncomp	comp		comp	n/comp	n/comp
	*K*_i_ (*K*_a_)	5.4	0.76	1.84		2.48	4.14	0.3

INH, inhibitor; AC, activator; comp, competitive; uncomp, uncompetitive; noncomp, noncompetitive; *K*_i_, inhibition constant, mM; *K*_a_, activation constant, mM; nd, not determined.

## Data Availability

All studied bacterial strains were deposited in the All-Russian Collection of Microorganisms (VKM) under their respective numbers. All genomic data discussed in the manuscript were deposited in publicly available databases, as stated above. Any additional data mentioned in the article are available from the authors upon request.

## Supplementary Materials

The following supporting information can be downloaded at: https://www.mdpi.com/article/10.3390/ijms25126409/s1.
