# Supplementary material for: Glyphosate-Induced Phosphonatase Operons in Soil Bacteria of the Genus Achromobacter
Source: Int J Mol Sci. 2024 Jun 10;25(12):6409. doi: 10.3390/ijms25126409 (PMC11203657; doi:10.3390/ijms25126409)
Supplement: Supplementary file 1 [file ijms-25-06409-s001.zip › ijms-2969439-supplementary.pdf]

Supporting information

1. Supporting tables

Table S1. Purification of phosphonatases from wild-type strains

Preparation	Purification stage	Protein, mg	Total activity, U	Specific activity, U mg^{-1} protein	Yield, %	Purification, fold
PhnX16	cfe*	255.0	0.459	1.8×10^{-3}	100	1
	Ion exchanger I	29.88	0.260	8.7×10^{-3}	56.7	4.8
	Gel filtration	0.24	0.122	516×10^{-3}	16.6	287
	Ion exchanger II	0.04	0.077	1716×10^{-3}	16.8	953
PhnX19	cfe	209.0	0.459	2.2×10^{-3}	100	1
PhnX19-I	Ion exchanger I	8.54	0.141	16.5×10^{-3}	28.9	7.5
	Gel filtration	0.06	0.086	1445×10^{-3}	18.8	656.8
	Ion exchanger II	0.02	0.056	2770×10^{-3}	12.1	1259
PhnX19-II	Ion exchanger I	0.91	0.073	80×10^{-3}	15.9	36.4
	Gel filtration	0.05	0.051	1062×10^{-3}	11.1	48.3
	Ion exchanger II	0.01	0.038	2550×10^{-3}	8.3	1159.5

* cfe, cell-free extract

Table S2. Purification of recombinant phosphonatases

Preparation	Purification stage	Protein, mg	Total activity, U	Specific activity, U mg^{-1} protein	Yield, %	Purification, fold
PhnX16-R	cfe*	101.60	1.615	15.9×10^{-3}	100	1
	Affinity chromatography	0.12	0.69	5760×10^{-3}	42.7	362.3
PhnX19-RI	cfe	76.50	6.5	85×10^{-3}	100	1
	Affinity chromatography	1.08	4.92	4557×10^{-3}	75.7	53.6
PhnX19-RII	Affinity chromatography	1.30	1.27	974×10^{-3}	19.5	11.5

* cfe, cell-free extract

Table S3. Genome assembly of *A. aegrifaciens* Kg 16 and *A. insolitus* Kg 19

Object	Genome size, mbp	Number of scaffolds	Largest scaffold, bp	N50, bp	G+C content, %	Coverage, ×
A. aegrifaciens Kg 16	6.643	40	1,189,585	507,084	66.07	723
A. insolitus Kg 19	6.438	43	973,458	628,760	65.19	630

2. Supporting figures

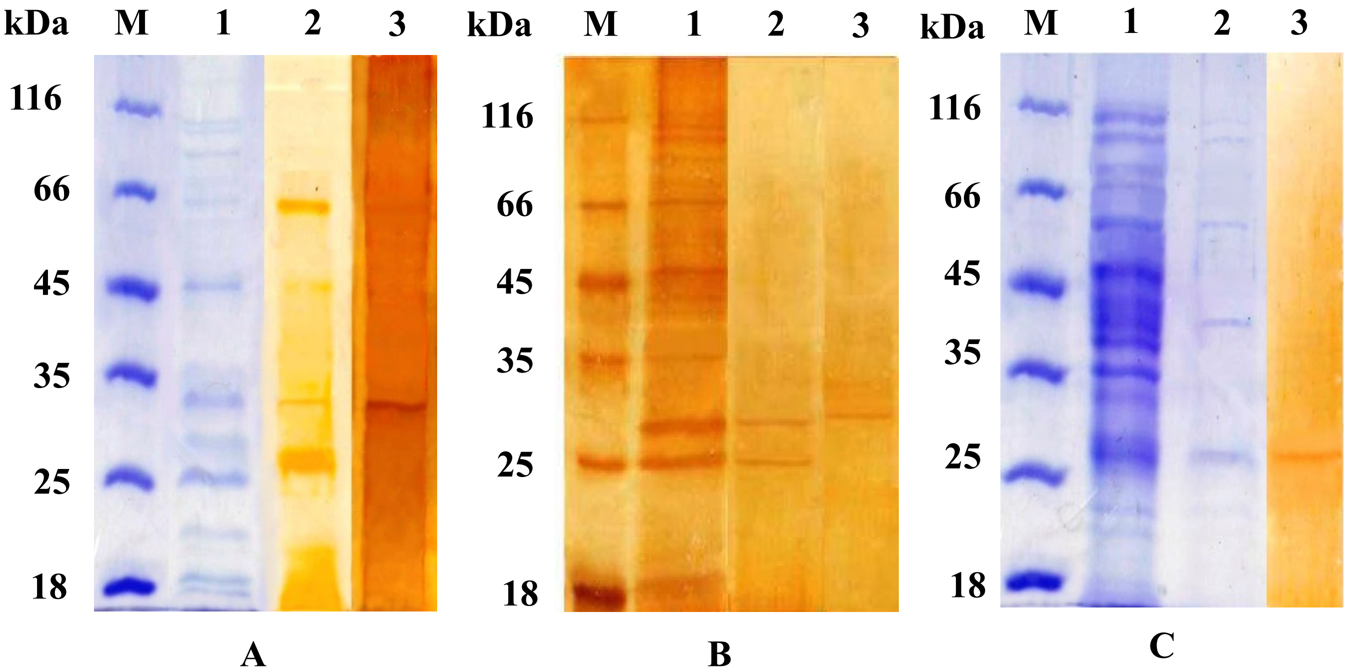

Figure S1. SDS-PAGE of PhnX19 (A), PhnX19-II (B) and PhnX16 (C) preparations after different purification stages: 1 – Ion Exchanger I; 2 – Gel filtration; 3 – Ion Exchanger II; M – Protein weight markers.

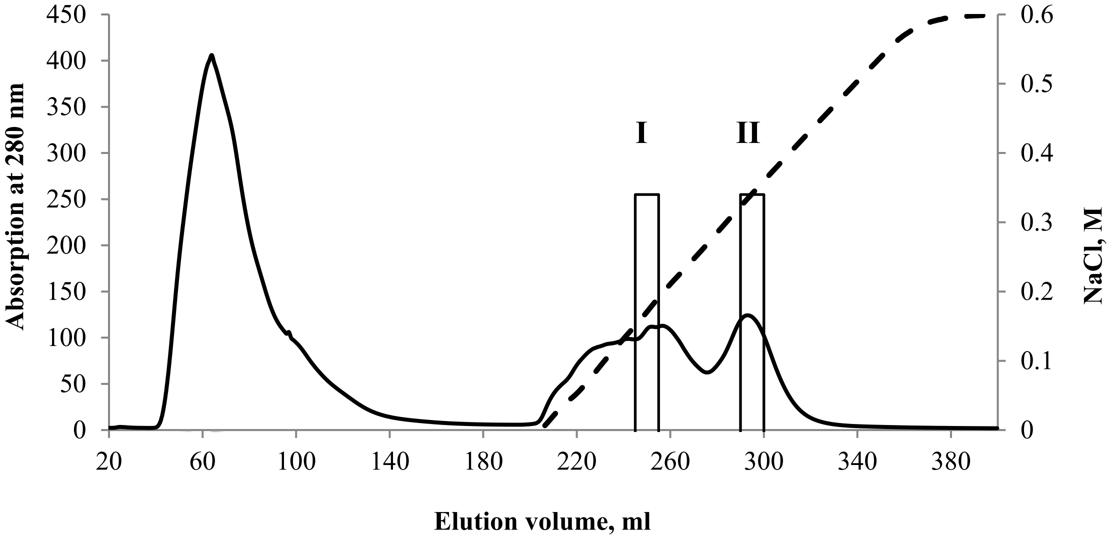

Figure S2. Elution profile of a cell-free extract of *A. insolitus* Kg 19 on a DEAE-Toyopearl column (16 × 700 mm). Solid line, absorption at 280 nm; dotted line, NaCl concentration; rectangles, fractions with phosphonatase activity designated as isoforms I and II

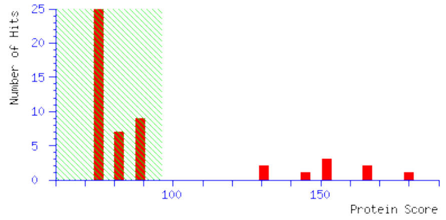

Protein sequence coverage: 79%

1	M**TVSPLPVRL**	**EAVIFDWAGT**	**LVDFGSFAPT**	**KVFVDAFSQF**	**GVEMSLAQAR**
51	**GPMGMGKWDH**	**IRTLCNDPVI**	**ASQYQAQFGR**	**LPGDDDVTAI**	**YERFLPMQLE**
101	**KVAQYSAAIP**	**GAAELLR**ALR	QRGLK**IGSCS**	**GYPASVMR**RV	VER**AASEGLE**
151	**PDCIVASDDV**	**PRARPAPAMA**	**LKNVIELGIS**	**DVAACVKVDD**	**TAPGIEEGRR**
201	**AGMWTVGLLL**	**SGNAAGLTLE**	**EYLSLDEAGR**	QQARTAASQE	LSPVAPHYLI
251	DTVADLPAVI	ADIESRLSAG	QRP		

A **B**

Figure S3. Results of MALDI-TOF analysis of PhnX19 and PhnX19-II. A – Mascot search histogram. Protein score is -10*Log(P), where P is the probability that the observed match is a random event. Protein scores greater than 96 are significant (p<0.05). Top score: 180 for OAD13647.1 (phosphonoacetaldehyde hydrolase, *Achromobacter insolitus*); B – Top score protein sequence coverage table. Matched peptides shown in bold.

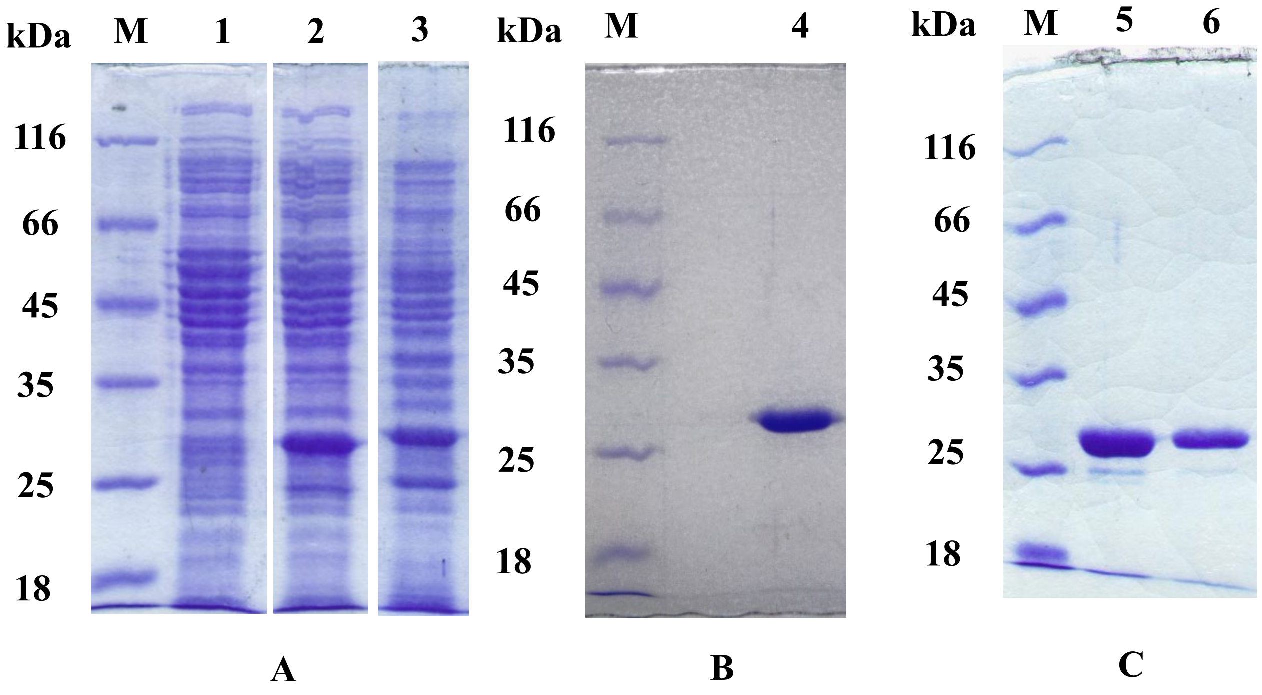

Figure S4. SDS-PAGE of cell-free extracts (A), PhnX16-R (B), PhnX19-R and PhnX19-RII (C): 1 – Negative control (*E. coli* BL21(DE3) after transformation with pET-22b vector without insertion); 2,3 – *E. coli* BL21(DE3) after transformation with pET-22b comprising *phnX16* and *phnX19* respectively; 4 – Purified PhnX16-R; 5 – Purified PhnX19-RI; 6 – Purified PhnX19-RII; M – Protein weight markers.

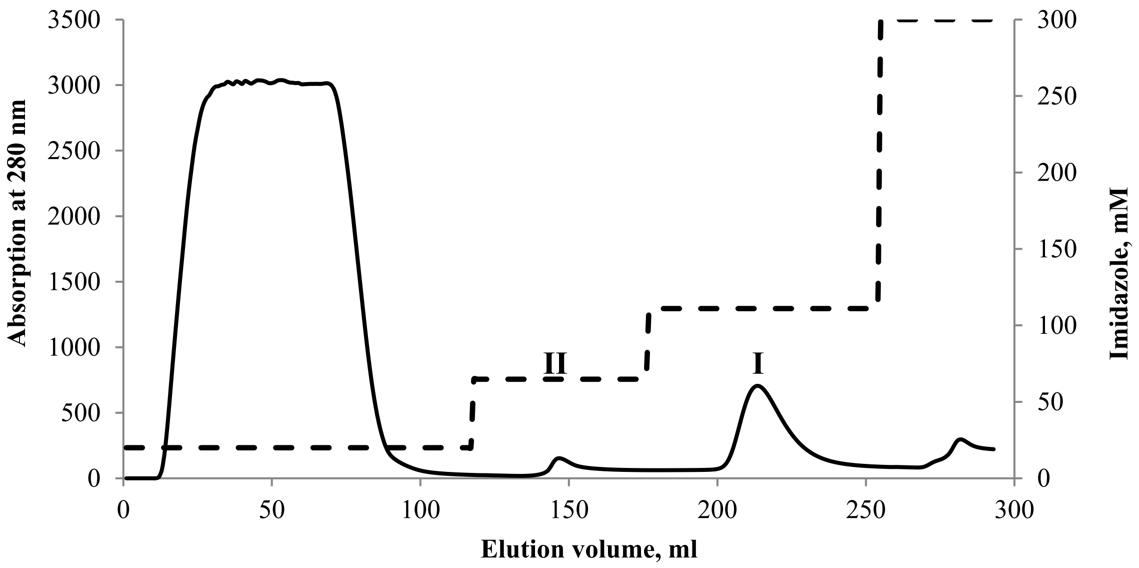

Figure S5. Elution profile of a cell-free extract of the PhnX19-R superproducing strain on a HisTrap FF column (5 ml). Solid line, absorption at 280 nm; dotted line, imidazole concentration; roman numerals, peaks with phosphonatase activity, respectively, PhnX19-RII and PhnX19-RI.

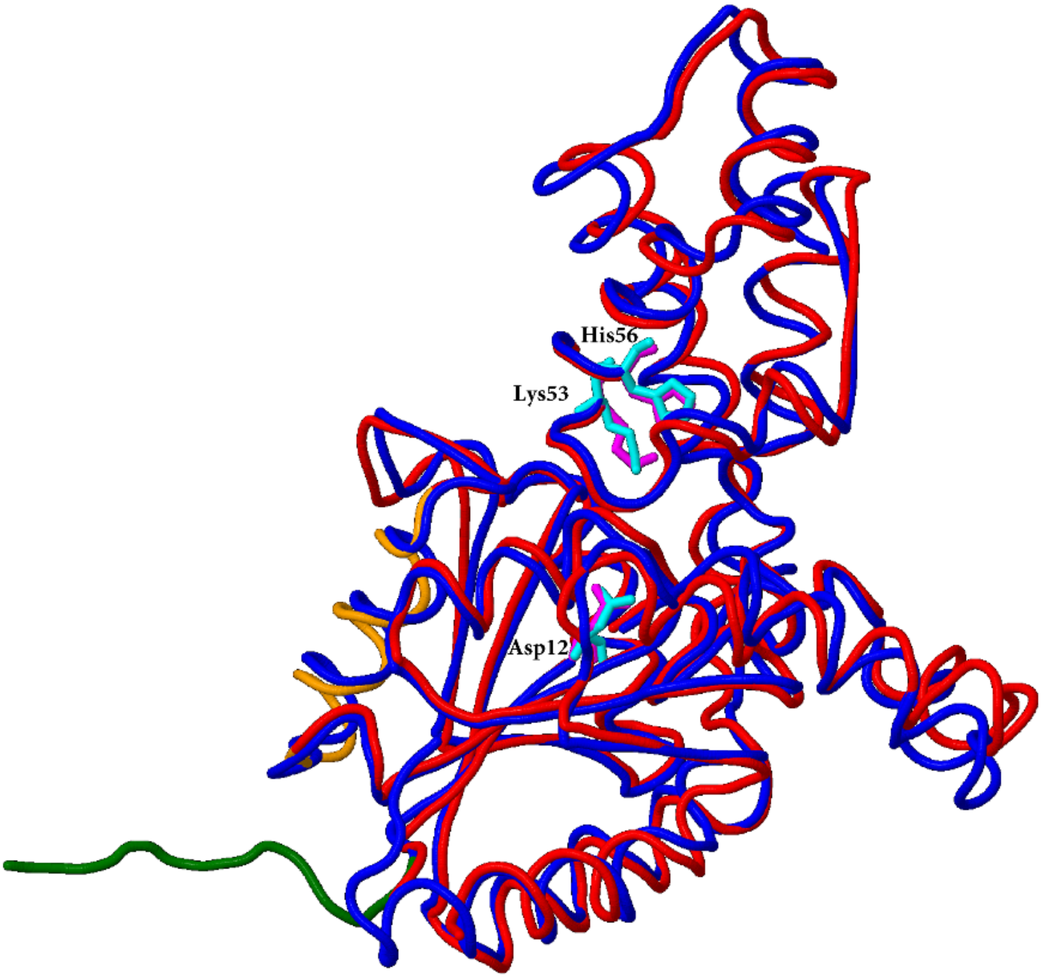

Figure S6. Superimposition of the predicted three-dimensional PhnX19 structure (blue) on the *B. cereus* phosphonatase model (red). Orange, the hydrophobic subunits' binding interface [32]. Green, the N-terminal sequence of PhnX19 with low reliability of structure prediction. Amino

acid residues directly involved in the catalysis from the side of the *cap* domain (Lys53, His56) and *core* domain (Asp12) are given in the form of structural formulae and are highlighted in color (magenta, *B. cereus*; turquoise, *A. insolitus* Kg19).

```
                                 1         10        20        30        40        50        60
                                 |         |         |         |         |         |         |
PhnX A. aegrifaciens Kg 16      MTVSPLPVRLEAVIFDWAGTLVDFGSFAPTKVFVDAFSQFGVEMSLAQARGPMGMGKWDH : 60
  PhnX  A. insolitus Kg 19      MTVSPLPVRLEAVIFDWAGTLVDFGSFAPTKVFVDAFSQFGVEMSLAQARGPMGMGKWDH : 60
            PhnX B. cereus      -------MKIEAVIFDWAGTTVDYGCFAPLEVFMAIFHKRGVEITAEEARKPMGLLKIDH : 53

PhnX A. aegrifaciens Kg 16      IRTLCNEPVIASQYQAQFGRLPTDDDVTAIYERFLPMQLDKVAQYSAAIPGAAELLRALR : 120
  PhnX  A. insolitus Kg 19      IRTLCNDPVIASQYQAQFGRLPGDDDVTAIYERFLPMQLEKVAQYSAAIPGAAELLRALR : 120
            PhnX B. cereus      VRALTEMPRIASEWNRVFGQLPTEADIHEMYEEFEEILFAILPRYATPIHGVKEVIASLR : 113

PhnX A. aegrifaciens Kg 16      QHGLKIGSCSGYPASVMRRVVERAATEGLEPDCIVASDDVPRARPAPAMALKNVVELGLS : 180
  PhnX  A. insolitus Kg 19      QRGLKIGSCSGYPASVMRRVVERAASEGLEPDCIVASDDVPRARPAPAMALKNVIELGIS : 180
            PhnX B. cereus      ESGIKIGSTTGYTREMMDIVEKEAAIQGYKPDFLVTPDDVPAGRPYPWMCYKNAMELGVY : 173

PhnX A. aegrifaciens Kg 16      DVAACVKVDDTAPGIEEGRRAGMWTVGLLLSGNAAGLTLEEYLSLDDAGRQKARTAASLE : 240
  PhnX  A. insolitus Kg 19      DVAACVKVDDTAPGIEEGRRAGMWTVGLLLSGNAAGLTLEEYLSLDEAGRQQARTAASQE : 240
            PhnX B. cereus      PMNRMIKVGDTVSDMKEGRNAGMWTVGVILGSSELGLTEEEVENMDPAELRERIEVVRNR : 233

PhnX A. aegrifaciens Kg 16      LSPAAPHYLIDTVADLPGVISDIEARLSAGQRP : 273
  PhnX  A. insolitus Kg 19      LSPVAPHYLIDTVADLPAVIADIESRLSAGQRP : 273
            PhnX B. cereus      FVENGAHFTIETMQELESVMEHIEKQELIIS-- : 264
```

Figure S7. Alignment of the amino acid sequences of phosphonatases from *A. aegrifaciens* Kg 16, *A. insolitus* Kg 19 and the previously described enzyme of *B. cereus*. Blue, amino acid residues known to form the active center of *B. cereus* phosphonatase [9]. Yellow, hydrophobic site of binding of the subunits in the dimer. Purple, amino acid substitutions that led to a decrease in the catalytic efficiency of *B. cereus* enzyme and are present in *Achromobacter* phosphonatases.

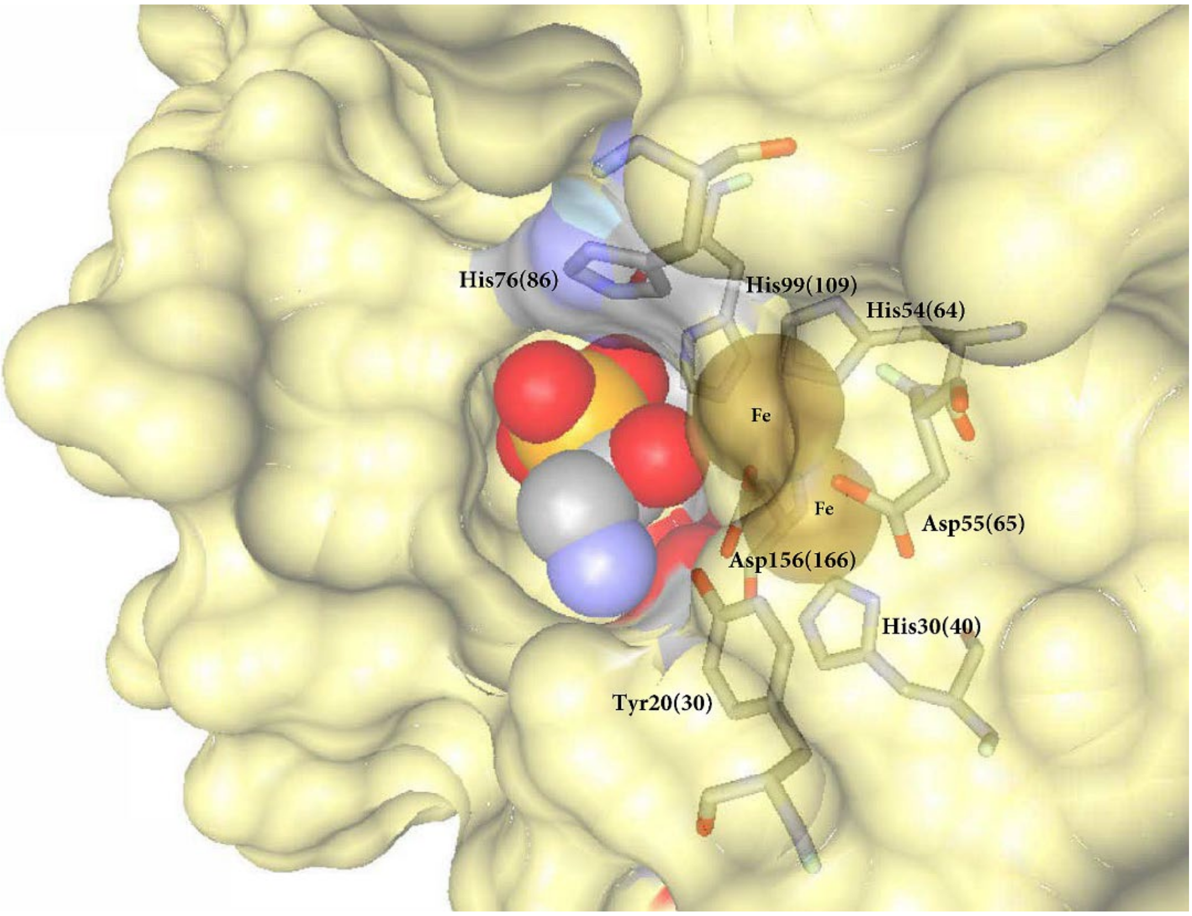

Figure S8. A model of the active center of *A. aegrifaciens* Kg 16 in the protein globule with a putative substrate (HAEP) bound in the catalytic cavity together with two iron ions. Red, oxygen; yellow, phosphorus; blue, nitrogen; brown, iron; gray, carbon. The position numbers of amino acids with a similar function in TmpB [12] are shown in parentheses.
